# Actionability and familial uptake following opportunistic genomic screening in a pediatric cancer cohort

**DOI:** 10.1038/s41431-024-01618-7

**Published:** 2024-05-13

**Authors:** Sophia Hammer-Hansen, Ulrik Stoltze, Emil Bartels, Thomas van Overeem Hansen, Anna Byrjalsen, Anne Tybjærg-Hansen, Klaus Juul, Kjeld Schmiegelow, Jacob Tfelt, Henning Bundgaard, Karin Wadt, Birgitte Rode Diness

**Affiliations:** 1 Department of Clinical Genetics, Copenhagen University Hospital Rigshospitalet, Copenhagen, Denmark; 2 Department of Pediatric and Adolescent Medicine, Copenhagen University Hospital Rigshospitalet, Copenhagen, Denmark; 3 Institute of Clinical Medicine, Faculty of Medicine, Copenhagen University Hospital Rigshospitalet, Copenhagen, Denmark; 4 Department of Clinical Biochemistry, Copenhagen University Hospital Rigshospitalet, Copenhagen, Denmark; 5 Department of Forensic Genetics, Copenhagen University Hospital Rigshospitalet, Copenhagen, Denmark; 6 Department of Cardiology, Copenhagen University Hospital Rigshospitalet, Copenhagen, Denmark

**Keywords:** Genetic testing, Population screening, Personalized medicine

## Abstract

The care for patients with serious conditions is increasingly guided by genomic medicine, and genomic medicine may equally transform care for healthy individual if genomic population screening is implemented. This study examines the medical impact of opportunistic genomic screening (OGS) in a cohort of patients undergoing comprehensive genomic germline DNA testing for childhood cancer, including the impact on their relatives. Medical actionability and uptake after cascade testing in the period following disclosure of OGS results was quantified. A secondary finding was reported to 19/595 (3.2%) probands primarily in genes related to cardiovascular and lipid disorders. After a mean follow up time of 1.6 years (Interquartile range (IQR): 0.57-1.92 yrs.) only 12 (63%) of these variants were found to be medically actionable. Clinical follow up or treatment was planned in 16 relatives, and as in the probands, the prescribed treatment was primarily betablockers or cholesterol lowering therapy. No invasive procedures or implantation of medical devices were performed in probands or relatives, and no reproductive counseling was requested. After an average of 1.6 years of follow-up 2.25 relatives per family with an actionable finding had been tested. This real-world experience of OGS grants new insight into the practical implementation effects and derived health care demands of genotype-first screening. The resulting health care effect and impact on demand for genetic counseling and workup in relatives extends beyond the effect in the probands.

## Introduction

Extensive germline genetic sequencing is currently used in both clinical practice and research studies, generating data also covering genes not related to the primary clinical question. The American College of Medical Genetics and Genomics (ACMG) has published a list of “actionable” genes, recommending universal reporting of known or expected disease-causing variants within these genes irrespectively of the clinical indication leading to germline sequencing, resulting in opportunistic genomic screening (OGS) for susceptibility to other preventable diseases in patients [1–4]. Previous studies of several cohorts have determined the rate of secondary genetic findings to be 1.0–3.4% [5–7]. However, within the genetic community the European Society of Human Genetics recommends a different approach, where OGS only is performed within a research setting generating more knowledge of genotype-first genetics [8].

Established clinical practice recommends cascade testing of a pathogenic (PV) or likely pathogenic (LPV) variant within a family for many of the genes listed as actionable by the ACMG. Testing at-risk individuals can provide tailored counseling and management of potentially life-threatening and treatable diseases [9]. As such, return of a secondary finding (SF) may lead to additional genetic consultations of both probands and relatives as well as genetic testing, clinical workup, treatment, and long-term follow-up of several individuals [7, 10]. While previous studies have described OGS findings in patients from patient cohorts or population genomic screening [7, 11–15] the down-stream impact that such a finding has on the index patient, as well as relatives requires further attention.

The aim of this study was to quantify the medical impact of OGS in a cohort of patients undergoing comprehensive genomic DNA testing for childhood cancer as well as the medical impact on their relatives. This was performed by quantifying actionability and uptake after cascade testing in the time following disclosure of OGS results to patients and relatives.

## Materials and methods

### Inclusion and exclusion

Participants (probands and parents), enrolled in the “**S**equencing of **T**umor **A**nd **G**ermline DNA – Implications and National Guidelines” (STAGING) study were included. The STAGING study is a Danish, prospective multicenter genomic sequencing study offering inclusion to children diagnosed with any cancer or CNS tumor before 18 years of age since January 1^st^ 2017 [16]. Patients were excluded from the STAGING study if they or the legal guardian did not consent to the return of an actionable genetic finding. Four-generation pedigrees were obtained.

### Sampling and variant interpretation

DNA sampling and sequencing of probands is described in supplementary methods. Variants were annotated and filtered using VarSeq (Golden Helix) as recently described [16]. Briefly, all variants with a minor allele frequency of >1% in any large population (gnomAD v2.1) were excluded. Only coding and splicing variants (+/− 10 bp) were included in the analysis. Moreover, only variants with a variant allele fraction > 20% were kept in the analysis. Finally, data was filtered for variants in 314 genes associated with cancer as well as the genes listed as actionable in the ACMG v.2.0 policy statement [2, 16]. This version of the ACMG policy statement was the recommended version when data was analyzed, and used throughout the study period to ensure continuity in data analysis, despite the publication of updated recommendations [4]. Only variants in ACMG genes, excluding cancer predisposing genes, were included.

Variants were assessed by a team of clinical geneticists and molecular biologists based on variant ontology (e.g., frameshift, nonsense, missense), in silico predictions of effect on protein and RNA function (e.g., Combined Annotation Dependent Depletion [CADD], PHRED quality score, ADA splice prediction score), and database searches for published literature on each variant. Health care records for the proband in some instances including an EKG or a lipid profile and a detailed pedigree was available to the interpreters. The ACMG guidelines regarding variant interpretation was not published when the study was planned, and not implemented at our institution when the first samples were analyzed [17]. During the study period the ACMG classification system was gradually implemented, and the previous local standard phased out. Only pathogenic or likely pathogenic variants were returned to the probands in accordance with the ACMG v.2.0 guidance. Challenging cases were discussed in special academic fora, see supplementary methods for details.

### Genetic counseling, clinical work up and cascade testing

Probands with a SF in one of the genes not associated with cancer predisposition on the ACMG v 2.0 list (36 genes), were informed of the results by a research team member and the findings were noted in the proband’s electronic medical record. The proband was referred to the local department of Clinical Genetics for clinical genetic workup and counselling, and if relevant, the proband/family was referred to clinical management with a relevant specialist (typically pediatrician or specialized cardiologist). Any clinical evaluation or counseling that was performed as result of the return of the SF was undertaken and funded by the Danish health care system and was not a part of the research protocol. Further clinical work up entailed obtaining a medical and family history, clinical exam, and further diagnostic testing such as imaging (echocardiography, cardiac and/or vascular MRI), EKG, 48-hour holter-monitoring, lipid profile measurements, assessment of ICD implantation risk, medical treatment initiation etc, at the discretion of the treating physician. See supplementary methods for further details. If relatives were identified as eligible for genetic counseling and/or cascade testing by the physician in charge of the clinical follow up in the proband, they were referred directly to genetic counselling in the relevant clinical specialty.

### Actionability and follow-up

Electronic medical records in probands and relatives were reviewed for family history, diagnostic testing results, referral to medical specialists, as well as medical treatment plans relating to the return of the SF. Actionability of a SF was defined as planned regular follow-up (e.g., lipid profile evaluation, clinical follow up in specialized cardiology or pediatric clinic, repeat cardiac imaging), prescription of medical or dietary treatment, invasive medical procedures, and risk reducing medical procedures (e.g., pacemaker or implantable cardioverter defibrillator implantations). Reproductive decisions due to the SF (prenatal diagnostic testing and preimplantation genetic testing) were also included. Uptake was defined as number of relatives tested for the variant per proband, when cascade testing was recommended for the disclosed SF variant.

### Statistics

Descriptive statistics were performed in Microsoft Excel (version 2016). Ninety-five percent confidence limits for point estimates were calculated by non-parametric bootstrap resampling in SAS (version 9.4).

### Ethical considerations

Ethical approval was obtained through the regional scientific ethical committee (the Ethical Scientific Committees for the Capital Region, H-15016782) and the Danish Data Protection Agency (RH-2016-219, I-Suite no: 04804). All parents/guardians and patients 18 years or older gave formal written consent to germline WGS in the proband as well as written, informed consent to the return of actionable findings as well as scientific reporting of this. Relatives beyond parents that were referred to genetic counselling or clinical workup due to a SF gave written consent to the inclusion of their data in a scientific publication.

## Results

### Secondary finding variants

**Fig. 1:**
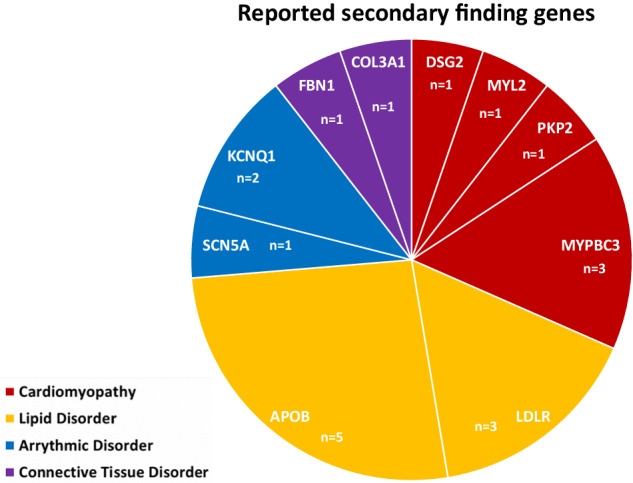
Distribution of genes reported with a secondary finding in ACMG version 2.0 genes not associated with cancer (36/59 genes). Findings are primarily related to genes related to cardiomyopathies or lipid disorders.

837 consecutive patients were invited to enroll in the STAGING study between January 1^st^ 2017 and December 31^st^ 2021, of which 665 patients consented and were included. At the time of analysis (April 2022), final data had been reported in 595 patients. Twenty-six pathogenic or likely pathogenic variants were detected in non-oncogene actionable genes in twenty-six probands, of which seven did not meet the gene-specific reporting criteria (heterozygosity of the recessive gene in question or likely pathogenic variant in a gene for which only known pathogenic should be reported), see Supplementary Table 1. Thus, a SF was returned to 19/595 (3.2% with 95% confidence limits 2.1%-4.2%) patients (Table 1). Of the 19 reported findings, seven were classified as PV and twelve as LPV using the aforementioned classification strategy, see Table 1. Variant interpretation using the ACMG guidelines is also noted in Table 1, further details on the criteria used can be found in supplementary table 2 Secondary findings were reported in 10 of the 36 SF genes, and primarily related to cardiovascular diseases (9/19 genes, Fig. 1), and in genes related to lipid disorders (8/19 genes) or connective tissue disorders (2/19).

**Table 1:** Reported secondary findings.

Family #	Gene	HGVS	HGVp	Variant classification at report	Variant classification after counseling	Variant classification (ACMG guidelines)^a^
Lipid Disorders						
4	*APOB* (NM_000384.2)	c.10580 G > A	NP_000375.2: p.(Arg3527Gln)	PV	PV	PV
10	*APOB* (NM_000384.2)	c.10808 A > G	NP_000375.2: p.(His3603Arg)	LPV	VUS	VUS
13	*APOB* (NM_000384.2)	c.11330 C > A	NP_000375.2: p.(Ser3777Ter)	LPV	^c^	LPV
15	*APOB* (NM_000384.2)	c.10580 G > A	NP_000375.2: p.(Arg3527Gln)	LPV	PV	PV
18	*APOB* (NM_000384.2)	c.10134delG	NP_000375.2: p.(Gln3378Hisfs^a^4)	LPV	^b^	LPV
12	*LDLR* (NM_000527.4)	c.1359-1 G > A	p.?	LPV	PV	PV
16	*LDLR* (NM_000527.4)	c.1238 C > T	NP_000518.1: p.(Thr413Met)	LPV	LPV	LPV
19	*LDLR* (NM_000527.4)	c.409 G > A	NP_000518.1: p.(Gly137Ser)	LPV	^b^	LPV
Cardiomyopathy Disorders						
1	*DSG2* (NM_001943.4)	c.918 G > A	NP_001934.2: p.(Trp306Ter)	PV	PV	PV
3	*MYBPC3* (NM_000256.3)	c.442 G > A	NP_000247.2: p.(Gly148Arg)	LPV	LPV	LPV
7	*MYBPC3* (NM_000256.3)	c.3628-41_3628-17del	p.?	PV	^b^	BV
14	*MYBPC3* (NM_000256.3)	c.3628-41_3628-17del	p.?	LPV	VUS	BV
11	*MYL2* (NM_000432.3)	c.403-1 G > C	p.?	LPV	LPV^d^	LPV
17	*PKP2* (NM_004572.4)	c.1643del	NP_004563.2: p.(Gly548Valfs^a^15)	PV	PV	PV
Arrythmia Disorders						
2	*KCNQ1* (NM_000218.2)	c.905 C > T	NP_000209.2: p.(Ala302Val)	LPV	LPV	PV
8	*KCNQ1* (NM_000218.2)	c.806 G > T	NP_000209.2: p.(Gly269Val)	PV	PV	LPV
5	*SCN5A* (NM_198056.2)	c.611+1 G > A	p.?	PV	PV	PV
Connective Tissue Disorders						
9	*COL3A1* (NM_000090.3)	c.2283+1 G > A	p.?	PV	LPV	LPV
6	*FBN1* (NM_000138.4)	c.6724 C > T	NP_000129.3: p.(Arg2242Cys)	LPV	VUS	VUS

*PV* pathogenic variant, *LPV* likely pathogenic variant, *VUS* variant of uncertain significance, *BV* benign variant.

^a^Richards et al. Genet Med, 2015. See Supplementary Table 2 for further details.

^b^No follow-up counseling.

^c^Truncating variants are associated with recessively inherited hypobetalipoproteinemia and not familial hypercholesterolemia.

^d^Only relevant if biallelic.

### Probands and genetic counseling uptake

The median age at return of the SF to the probands was 13.4 years (inter quartile range (IQR): 6.15–17.2 yrs), and median follow-up time since return of SF was 1.6 years (IQR: 0.57–1.92 yrs). One patient was referred to genetic counseling but did not attend the planned session, two cases were handled by the treating oncological pediatrician and the family did not wish further genetic consultation, but the remainder (*n* = 16) received genetic counselling and/or referral to relevant specialist (cardiologist or specialized pediatrician). One proband died before the return of the SF, which subsequently was reported to the parents, who were referred to genetic counseling. In nine families there was a suggestive family history of a relevant cardiovascular disease, most commonly of a lipid disorder, but without previously identified genetic or clinical diagnosis in the family.

### Clinical actionability

**Fig. 2:**
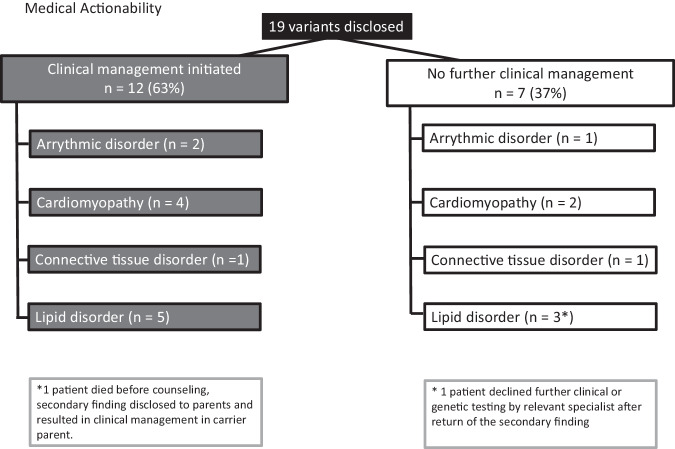
Actionability of disclosed variants by disease category after clinical and/or genetic workup in proband and relatives.

Clinical workup of the probands or families in which a SF was reported led to ongoing changes in clinical management in eleven of the living probands, and in the one family where the proband died before genetic counseling (Fig. 2). Further details regarding actionability can be found in Table 2 No invasive procedures or implantation of medical devices were performed in probands or relatives, and no reproductive genetic counseling or testing was requested. On average, 2.25 family members underwent genetic testing per proband in the families with actionable findings (27 relatives from 12 families). Family pedigrees are found in Supplementary Fig. S1.

**Table 2:** Actionability and clinical consequences for probands and relatives.

Family^e^	Age^a^, yrs	Variant	Condition	Proband symptoms^b^	Actionability proband	Previous family history^c^	Clinical consequence for relatives
**Lipid disorders**							
4	19.6	*APOB:*c.10580 G > A, p.(Arg3527Gln)	Familial hypercholesterolemia	Elevated cholesterol levels.	Regular follow-up in lipid clinic with dietary management while statin treatment is contraindicated during treatment with posaconazol post stem cell transplant.	Mother and grandparents with elevated cholesterol levels, no previous diagnosis of familial hypercholesterolemia.	Genetic testing of FDG already in statin therapy, carries variant. Seen in lipid clinic, target LDL set lower than previously.
							1 FDR, 2 SDR, informed, cascade testing is ongoing.
10	17.8	*APOB:*c.10808 A > G, p.(His3603Arg)	Familial hypercholesterolemia	None.	Lipid profile in patient and segregation analysis as part of genetic counseling resulted in reclassification of variant.	Elevated cholesterol levels in mother and paternal grandfather. Ischemic cardiovascular disease in paternal grandfather and maternal grandmothers’ sibling. No previous diagnosis of familial hypercholesterolemia.	Segregation analysis and lipid profile in 2 FDR to clarify clinical significance of variant.
					No additional treatment or follow-up.		Elevated Lp(a) detected in 1 FDR and recommended follow-up.
13	5.4	*APOB:*c.11330 C > A, p.(Ser3777Ter)	Familial hypercholesterolemia	No clinical testing performed	No additional testing or follow-up after genetic consultation and reclassification.	Premature cerebrovascular disease in maternal grandmother. No previous genetic diagnosis in the family.	Lipid profile in FDR, found to be low, no additional workup.
							Lipid profile testing in FDR, recommended follow-up with general practitioner due to increased cholesterol.
15	17	*APOB*:c.10580 G > A, p.(Arg3527Gln)	Familial hypercholesterolemia	Elevated cholesterol levels.	Statin treatment prescribed, and regular follow-up started at lipid clinic.	Family history of colorectal cancer with early presentation. No prior diagnosis of familial hypercholesterolemia.	Genetic testing of 2 FDG, both non-carriers.
					Recommended additional genetic consult at age 25 due to family history of colorectal cancer with early debut. ^e^		SDR informed, cascade testing is ongoing.
18	6.0	*APOB:*c.10134delG, p.(Gln3378Hisfs^a^4)	Familial hypercholesterolemia	None. Normal lipid profile.	Recommended check-up as an adult.	None	Normal lipid profile in one FDR.
							Reevaluation recommended in one FDR due to slight increase in LDL.
12	14.1	*LDLR:*c.1359-1 G > A, p.?	Familial hypercholesterolemia	Elevated cholesterol levels.	Statin therapy prescribed.	Mother, maternal uncle and maternal grandmother with elevated cholesterol levels and medical therapy. No prior diagnosis of familial hypercholesterolemia in the family.	Genetic testing of 1 FDG already in statin therapy for many years, carries variant. Add-on therapy prescribed (Ezetemibe) to reach LDL target. Regular follow-up planned in lipid clinic.
					Regular follow-up in pediatric lipid clinic.		1 FDR tested, found to be carrier with elevated LDL levels, statin therapy and regular follow-up in lipid clinic initiated.
							Two SDR already on statin therapy were informed but not yet tested. Cascade testing is ongoing.
16	15.2	*LDLR*:c.1238 C > T, p.(Thr413Met)	Familial hypercholesterolemia	Elevated cholesterol levels.	Dietary management started, reevaluation regarding statin therapy planned.	Elevated cholesterol levels in maternal grandfather and maternal aunt. No prior diagnosis of familial hypercholesterolemia.	1 FDR tested, carries the variant. Statin therapy prescribed
					Regular follow-up in pediatric lipid clinic.		1 FDR with low LDL levels, genetic testing not performed.
							Cascade testing is ongoing.
19	3.5	*LDLR:*c.409 G > A, p.(Gly137Ser)	Familial hypercholesterolemia	None. Normal lipid profile.	No further follow-up planned.	Elevated cholesterol levels in mother’s grandparents and great grandparent, not specified further. No prior diagnosis of familial hypercholesterolemia.	1 FDR actively declined additional testing or counselling.
**Cardiomyopathy Disorders**							
1	19.3	*DSG2:*c.918 G > A, p.(Trp306Ter)	Arrhythmogenic right ventricular cardiomyopathy	None. Workup in outpatient cardiology clinic: normal EKG, echocardiography, holter monitoring.	Regular follow-up in specialized cardiology clinic planned in three years.	None.	Cascade testing is ongoing.
3	17.4	*MYBPC3*:c.442 G > A, p.(Gly148Arg)	Hypertrophic cardiomyopathy	Echocardiography with discrete mitral regurgitation but no cardiomyopathy. Normal EKG and holter monitoring.	Regular follow-up in specialized cardiology clinic planned in five years.	None.	1 adult FDR tested, carries the variant. Skin biopsy of FDR performed for further variant classification with RNA analyses. Analysis not possible; gene not expressed in blood or skin fibroblasts. Echocardiography: unclear phenotype, Cardiac MRI: findings indicative of hypertrophic cardiomyopathy. Normal EKG and holter. Regular follow-up with cardiac evaluation including echocardiography and holter (planned in 1.5 years).
							1 FDR tested, carries the variant. Cardiac evaluation (clinical, echocardiography and EKG): no hypertrophic cardiomyopathy phenotype. Regular follow-up in specialized cardiology clinic planned in five years.
							Cascade testing is ongoing.
7	13.4	*MYBPC3*:c.3628-41_3628-17del, p.?	Hypertrophic cardiomyopathy	n/a, patient has not sought genetic counseling.	Pediatric oncology evaluation: schedule MUGA scan every three years due to risk of cardiomyopathy.	None.	FDRs have not actively sought genetic counseling.
14	14.6	*MYBPC3:*c.3628-41_3628-17del, p.?	Hypertrophic cardiomyopathy	No clinical evaluation performed.	Variant reclassified after genetic counseling, no further testing.	Paternal grandfather with heart disease, not specified further.	No testing in family.
11	7.6	*MYL2:*c.403-1 G > C, p.?	Hypertrophic cardiomyopathy	No clinical evaluation performed.	No further testing or follow-up after genetic consult as clinically relevant disease only predicted in patients with biallelic variants.	Premature ischemic cardiovascular disease in maternal grandfather and his father. No prior genetic diagnosis in family.	One FDR and her two siblings recommended lipid profile (primary care) due to family history of premature cardiovascular disease.
17	3.7^d^	*PKP2:*c.1643del, p.(Gly548Valfs^a^15)	Arrhythmogenic right ventricular cardiomyopathy	Not available, patient deceased before clinical evaluation.	n/a, deceased when genetic testing reported.	None.	Testing of 2 FDR, one carries the variant.
							Clinical workup of carrier FDR with EKG, echocardiography, holter monitoring. Asymptomatic, annual follow-up in specialized cardiology clinic.
							3 SDR were informed but did not wish further testing or follow up.
							Testing of TDR with normal result.
**Arrythmia Disorders**							
2	8.8	*KCNQ1:*c.905 C > T, p.(Ala302Val)	Long QT syndrome	Asymptomatic.	Prescribed treatment with beta blockers.	None.	Genetic testing of 4 FDRs. One asymptomatic carrier (QTc normal range) evaluated in cardiology outpatient clinic: normal echocardiography, prescribed betablockers. Yearly follow up in cardiology clinic.
					Yearly follow-up in cardiology clinic.		2 SDR tested, one carrier.
					Extra attention when treating with certain QT-prolonging agents and periods of hypokalemia.		1 TDR tested, non-carrier.
8	18.5	*KCNQ1:*c.806 G > T, p.(Gly269Val)	Long QT syndrome	QTc abnormal upon cardiac evaluation.	Prescribed beta blockers.	None.	Genetic testing of 3 FDRs, all non-carriers.
					Yearly follow-up in cardiology clinic.		1 FDR referred to genetic counseling at local clinic due to family history of breast cancer. Conclusion of evaluation: moderately increased risk of breast cancer, recommended yearly clinical mammography age 40-50 years.
					Extra attention when treating with certain QT-prolonging agents and periods of hypokalemia.		
5	6.3	*SCN5A:*c.611+1 G > A, p.?	Brugada syndrome	Normal EKG.	,No further follow-up planned.	None.	normal EKG in 2 FDR, no genetic testing or additional follow-up planned (shared decision making with family).
**Connective Tissue Disorders**							
9	6.5	*COL3A1:*c.2283+1 G > A, p.?	Vascular Ehlers-Danlos syndrome	Dysmorphic features: thin vermillion of the lips, narrow nose, prominent eyes. Finger and thumb hypermobility. Bilateral pes valgus. Echocardiography performed without abnormal findings.	Yearly follow-up in specialized pediatric center for rare diseases (already seen here due to neurofibromatosis 1 diagnosis).	Abdominal aorta aneurisme cause of death in great grandfather age 90 yrs. No prior genetic in the family.	1 FDR found to carry variant. Scheduled yearly follow-up in specialized center for rare diseases. Yearly cardiology follow-up in outpatient clinic initially, betablocker treatment prescribed. Now biennial follow-up with cardiac MRI (normal tests). Skin biopsy and fibroblast culture performed for further variant classification with functional assay resulting in C4 classification.
							Predictive testing of 3 SDR, one confirmed heterozygous and referred to specialized center with follow-up in cardiology and specialized center for rare diseases as well as prescription of betablocker.
							2 TDRs tested, found negative. Cascade testing is ongoing.
6	3.1	*FBN1:*c.6724 C > T, p.(Arg2242Cys)	Marfan syndrome	No clinical evaluation performed.	Variant not diagnostic of Marfan syndrome after genetic counseling and work up of adult FDR. No further follow-up or treatment of proband.	None.	Testing of 2 FDR to clarify if variant was de novo.
							Clinical workup for Marfan syndrome of heterozygous adult FDR: normal ophthalmological evaluation, normal echocardiography, normal cardiac MRI. Clinical genetics evaluation with Marfan syndrome systemic score of 5 points. The variant was reclassified as ACMG VUS; clinical evaluation supported non-pathogenicity

*FDR* first degree relative, *SDR* second degree relative, *TDR* third degree relative, *LDL* low densitliy poprotein, *Lp(a)* lipoprotein(a).

^a^At report of secondary finding.

^b^At clinical workup.

^c^Disclosed at time of enrollment in STAGING study.

^d^Deceased at report of secondary finding

^e^Not related to patient’s cancer diagnosis, Hodgkin’s lymphoma

### Lipid disorders (*n* = 8)

Five of the variants disclosed in which actionable therapy was planned were in *LDLR* and *APOB*, genes which are associated with lipid disorders. Statin therapy or dietary treatment was prescribed after evaluation in a specialized lipid clinic in four probands, and further follow up in adulthood has been planned in the fifth proband. In one of these probands, who harbored a pathogenic *APOB* variant, the prescription of statin treatment was complicated by the fact that these drugs are contraindicated during posaconazol treatment, prescribed as fungal prophylaxis as pediatric oncological care. While cascade testing is ongoing, statin therapy was initiated or add-on lipid lowering therapy was prescribed in four relatives found to be a carrier (one a sibling), as well as regular appointments in the lipid clinic. In families 13 and 10, segregation analysis and lipid profiles of the parents resulted in reclassification of the variants and no additional treatment. The probands in the two families that were handled by the treating oncology pediatrician (family 18 and 19) did not have elevated lipid levels. Lipids profile testing was offered to these parents: reevaluation was recommended in one parent due to slightly elevated LDL levels, and in one family additional testing or counselling was actively declined.

### Cardiomyopathy disorders (*n* = 6)

Two probands (family 1 and 3) underwent clinical work up in the outpatient cardiology clinic after *DSG2* and *MYBPC3* variants were reported: no cardiomyopathy was detected by echocardiography, EKG and holter-monitoring were also normal, and there was no relevant family history or reported symptoms, why regular follow-up is planned in 3–5 years. Cascade testing revealed an asymptomatic carrier parent in family 3 with normal EKG and holter-monitoring. Echocardiography in this individual was of an unclear phenotype, but at diagnosis of hypertrophic cardiomyopathy made after cardiac MRI, due to indicative imaging findings. Regular follow up with cardiac evaluation is planned. A healthy sibling also underwent genetic testing in this family, after diagnosis in the parent, and found to also carry the *MYBPC3* variant. Cardiac evaluation (clinical exam, echocardiography and EKG) did not reveal a hypertrophic cardiomyopathy phenotype; a follow up in the specialized cardiology clinic is planned in 5 years. In family 17, the *PKP2* variant was reported to the parents, who both underwent genetic testing. Subsequently, clinical workup of the asymptomatic carrier parent entailing echocardiography, holter-monitoring, and EKG was performed. These exams and medical history were normal in the carrier parent; annual checkups in a specialized cardiology clinic are planned. Four cases of sudden death were reported in this family, in the parent’s third- and fourth-degree relatives, but it was not possible to attribute these deaths to *PKP2* carrier status after testing three relatives in this family. Interestingly, three relatives in this family actively declined genetic testing. The proband in family 7 (*MYBPC3*), did not attend genetic counselling. Despite this, the pediatric oncologists have planned muligated acquisition (MUGA) scans every three years due to the perceived risk of dilated cardiomyopathy as a carrier of a *MYBPC3* variant, after reading the results of the SF in the medical records. No further clinical testing or clinical management was necessary in the proband or family following genetic consultation in family 11 (*MYL2*) and family 14 (*MYBPC3*), due to reclassification of the variant as a variant of unknown significance (VUS), or because the variant was only clinically relevant if biallelic. In total, five at-risk-relatives were tested for SF variants in cardiomyopathy genes, of which three are carriers, and cascade testing is ongoing in two families.

### Arrythmia disorders (*n* = 3)

In the two probands (from family 2 and 8) with *KCNQ1* variants (associated with long QT syndrome), medical treatment with betablockers was prescribed by the treating cardiologist as well as annual follow up in a specialized cardiology clinic. As part of the counseling, information was given specifically to avoid hypokalemia and QT prolonging drugs. Cascade testing of ten at risk relatives identified two carriers of which data only was available from one, in whom betablocker therapy was prescribed despite normal EKG and echocardiogram. Thus, betablocker therapy was initiated in both asymptomatic patients and carrier relatives with no family history of sudden death, but as carriers of disease causing variants, as is recommended clinical practice [18, 19]. A shared decision-making strategy was planned in family 5 (*SCN5A*) with the clinical geneticist and cardiologist. The family was offered full clinical work up (ajmaline challenge to variant carriers and advice regarding possible triggers), requiring the family to commute to a university hospital. There was no relevant family history of arrhythmic disorders and based on the situation in their family, the parents chose only to have tests done that could be done by the local general practitioner. The family’s wishes of no further referrals was respected. They have the option of referral at a later time, should their wishes change. As such, Family 2 and 8 had a similar offer and weighed their options differently. The fact that a pharmaceutical intervention to ameliorate risk is available for patient with LQTS but not in Brugada syndrome may have influenced their decision, but this has not been explored in depth.

### Connective tissue disorders (*n* = 2)

The diagnosis of vascular Ehlers-Danlos syndrome (*COL3A1*), was made in a proband and two of the six tested relatives. These individuals were referred to regular clinical follow-up in a specialized pediatrics center for rare diseases where a dedicated team manages syndrome-related issues such as cardiovascular follow up. Baseline echocardiography in the proband and carrier relatives were normal. Regular checkups at a specialized cardiovascular clinic with annual cardiovascular MRI scans for the adults and echocardiograms in the child are planned. Treatment with the specific betablocker celiprolol has been prescribed in both adult carriers. For the proband in family 6 (*FBN1* LPV) testing of the parents was undertaken. The mother carried the reported variant and was extensively examined for Marfan syndrome phenotype (ophthalmological evaluation, echocardiography, cardiac MRI, clinical evaluation of Marfan systemic score) and a medical and family history were obtained. She did not fulfill the diagnostic clinical criteria, resulting in reclassification of the variant as a VUS, and no further clinical testing or treatment was warranted in the proband or relatives.

### Genetic counseling issues

While cascade testing is ongoing in seven families (median time since return of SF 1.89 years, IQR 1.63-2.02 yrs), we are aware of four relatives, from two families, who actively declined genetic testing (family 17 and 19). In one family, relatives initially declined genetic testing due to fear that non-paternity unknown to the relatives’ children would be disclosed. Additional genetic consultation of the mother in family 8 was planned at the local genetics department after the initial genetics consult regarding the SF, due to a detected familial risk of breast cancer that was independent of the proband’s disease and SF. Analysis of a panel of breast and ovarian cancer genes in the mother was performed with normal results, why she was recommended annual mammographies due to the moderately increased risk of breast cancer. Similarly, five relatives were recommended additional evaluation, due to a family history of ischemic cardiovascular disease or slightly elevated lipid levels in a non-carrier parent that came to attention during the genetic counseling pertaining the unrelated SF. When these individuals are accounted for, additional follow up or treatment was planned in a total of 16 relatives because of both cascade testing and “downstream” genetic counseling initiated by the return of the SF. While most relatives were tested due to cascade testing (*n* = 27), eleven relatives were tested or underwent medical evaluation such as segregation analysis.

## Discussion

This study elucidates the impact OGS of actionable genes has on patients and relatives in a country with free nationwide health care. The SF rate of 3.2% is higher than previously reported, e.g. by Haer-Wigman et al., in which the detection rate in 1640 healthy individuals was 1.5% in cardiovascular and connective tissue genes [20]. This may be due to the smaller sample size in the current study or differences in population frequencies of certain diseases, such as lipid disorders, which are more prevalent in northern Europeans (1/137 for familial hypercholesterolemia in Denmark) and not reported in the Haer-Wigman paper [21, 22]. A recent study from Germany reported a frequency of SF (2,6%) which is within the confidence limits in the current study, though this study used the ACMG version 3.0 SF list, and did not report lipid disorders [23]. Differences in variant interpretation may also play a role: if only pathogenic variants were reported, the rate would have been 1.2%, which is more in line with previous reports [6]. In this paper, only findings in non-cancer genes are reported, which makes the findings less generalizable, as many (23/59) of the actionable genes are cancer genes. Also, the population has low rates of consanguinity, hence the prevalence of recessively inherited disorders may be higher in other populations.

The uptake of cascade testing was 2.25 pr index patient during an average of 1.6 years of follow up. In seven families, cascade testing is ongoing, why this number will rise. Frey et al. studied uptake of cascade testing in a cohort of relatives to patients with pathogenic variants in hereditary cancer syndromes and similarly found an average of 1.9 relatives pr index case over two years [24]. This underscores that in a public health care setting with easy access to genetic counseling, the effect of disclosing an actionable variant extends beyond the effect for the index case.

In the probands, uptake to genetic counseling or clinical testing for the SF was high (84%, 16/19). We are aware of four relatives that actively declined genetic testing. It is a weakness of our design that we cannot distinguish between relatives that have actively declinedand relatives that simply have not yet been tested. In a recent review of genetic testing in relatives from families with hereditary cancer, uptake was found to be 48% (95% CI 38-58) overall [25]. In a similar meta-analysis of relatives in families with hypertrophic cardiomyopathy uptake varied from 37% to 84% [26]. It appears that uptake was high in our cohort. We propose that this is due to the direct contact with the families and the accessibility of health care in Denmark. Families that have experienced pediatric cancer may be less worried about the risk of cardiovascular disease. Conversely, the families’ behavior and responses may be atypical due a challenging situation with either on-going cancer treatment, terminal disease, or follow-up in survivors. Also, actionability of SFs may be limited by other health issues until later, such as the case of the delayed statin therapy due to concomitant antifungal therapy. Uptake could possibly be facilitated by providing letters to the families explaining the actionable finding and recommended follow-up.

Medical actionability is a key argument for implementation of OGS and the return of SFs. In this study, medical interventions such as regular follow up, dietary or pharmacological treatment were initiated in 61% of probands with a SF, as well as in 17 relatives. In five families, the patients and/or relatives had a phenotype consistent with the SFs, and typically a lipid disorder (families 4, 12, 9, 15 and 16). In one family (family 3, *MYBPC3*) the phenotype was not quite as clear- only findings on cardiac MRI in one adult carrier were consistent with hypertrophic cardiomyopathy. Interestingly, additional medical management was initiated in asymptomatic individuals on basis of the variant (families 1, 2, 8, 17 and 18) in five families. In the current study, all genetic results including SFs, were documented in the electronic medical record. In this setting, information is accessible to the patient and medical staff, not only researchers. In family 7, where a *MYBPC3* variant was reported, but the family did not seek genetic counseling. Thus, reclassification to a benign variant based on ACMG criteria was not changed accordingly in the medical records, resulting in pediatric assessment of a need for additional MUGA scans. Conversely, knowledge of an actionable finding that isn’t acted upon may create ethical or legal issues, which may be elucidated by further research.

Several families reported a family history of cardiovascular disease, but not a previous diagnosis of hereditary disease. This finding emphasizes the fact that SFs can be expected to be disclosed to families unaware of a risk of inherited disease. Counseling in these families is complicated by paucity of penetrance estimates for many diseases in patients without relevant family history, which is highlighted by the fact that several of the carriers of a SF were asymptomatic in this study [27]. Neither probands nor relatives underwent invasive procedures or the implantation of medical devices. This could be expected to be different if cancer predisposition genes such as *BRCA1* or *BRCA2* were included in the return of SFs, as in a recent paper from the BabySeq project, where the return of a genomic finding in children led to risk reducing surgeries in parents [15].

The risk of burdening patients with concerns that later are unfounded is a general argument against screening [8]. In this study 3 variants were reclassified from LP to VUS as a result of the clinical work up and genetic counseling with implementation of ACMG variant interpretation. Since the development of this study the ACMG guidelines on variant interpretation have become widely accepted and implemented. This development, along with the increased access to variant databases and ongoing work from ClinGen to provide gene specific classification guidelines (e.g. for *FBN1*), may reduce the probability of variant misclassification in the future [28]. Variant interpretation is a common challenge, both in diagnostic testing as well as in a setting of OGS as reported by Hart et al., where 14 variants of the 76 reported variants (18%) were reclassified as a VUS after evaluation of new evidence [7]. An example of variant misclassification in this study is the reported variant APOB: c.11330 C > A, p.(Ser3777Ter), family 13. This variant was mistakenly classified as a likely pathogenic variant associated with familial hypercholesterolemia type 2 (OMIM# 144010) at the time of reporting, while truncating *APOB* variants are associated with hypobetalipoproteinemia (OMIM # 615558). Difficult cases were discussed in the academic fora we described, but this variant was not considered there. In hindsight this error highlights that it would have been preferable to discuss all variants in a routine fashion before reporting. Careful variant interpretation is especially important in the setting of SFs and should be undertaken according to best available evidence. Further research in the related psychological and emotional impact in these individuals will be interesting.

While the ACMG position statement provides guidance for actionability in a setting of OGS, certain genetic variants may be more or less “actionable” in different populations or patient cohorts. For example, patients with Charcot-Marie-Tooth disease due to duplication of the *PMP22* gene are at higher risk of permanent neurological complications after treatment with certain antineoplastic agents [29]. Knowledge of such a predisposition could potentially be useful when planning oncological treatment. Recently, the list of medically actionable genes was expanded to 81 genes, and could potentially grow after each version, as more knowledge of genotype-phenotype relationships are understood and novel medical therapies become available [3, 4, 30]. As reported by Johnson et al., the returnable variant frequency rate increased by 22% when 73 actionable genes from the third version of the ACMG actionable list were analyzed compared to 59 genes from the second version [31]. If implemented, this can be expected to increase the work load on both variant interpretation laboratories and genetic counseling resources as well as impact more patients and their families. Likewise, it is helpful to have strategies in place, such as academic fora with expertise within different specialties (cardiovascular genetics, genetic oncology etc), to ensure that the management of such secondary findings happens in a standardized manner, while taking into account the specifics of each case such as shared decision-making strategies.

## Conclusion

In conclusion, OGS of 595 pediatric cancer patients resulted in the disclosure of a SF to 19 probands (3.1%) and led to clinical workup and genetic counseling in 38 relatives and planned follow up or treatment in 27 individuals. The number of relatives from this cohort offered medical intervention is expected to rise since cascade screening is ongoing. If OGS is undertaken, the resulting health care impact and demand for genetic counseling and workup in relatives extends far beyond the effect in the probands. This real-life experience further emphasizes the need for robust set-ups to ensure expert contribution and clinical consensus from clinicians in all specialties and institutions to care for both patients and relatives. Frameworks for the return of SFs are important to ensure systematic approaches and platforms for the return of such findings [10, 32, 33]. Finally, research in the patient experience, both when correctly, and incorrectly identified as a person at risk of an inherited disorder is warranted, for example via semi-structured interviews of the relatives involved in this study. This study demonstrates the feasibility of genetic screening and illustrates that the health care system in our setting can effectively handle the resulting needs for care in these families.

### Supplementary information

Supplementary methods

Table S1

Table S2

Figure S1

Supplementary Figure S1 Legend

## Data Availability

Data analyzed during this study can be found within the tables, figures, and supplementary materials.
