# Supplementary material for: Actionability and familial uptake following opportunistic genomic screening in a pediatric cancer cohort
Source: Eur J Hum Genet. 2024 May 13;32(7):846–57. doi: 10.1038/s41431-024-01618-7 (PMC11220050; doi:10.1038/s41431-024-01618-7)
Supplement: Supplementary file 1 — Supplementary methods [file 41431_2024_1618_MOESM1_ESM.docx]

Supplementary Methods:

DNA sampling and sequencing:

DNA sampling and sequencing of probands in the STAGING study has previously been described.^1^ Parents were not routinely sequenced. In short, genomic DNA was isolated from peripheral blood samples or, rarely, skin biopsies. Whole genome sequencing (WGS) was performed using the HiSeqX platform (Illumina, San Diego, CA, USA) with paired-end sequencing of 150-bp reads with a target of 30X in mean coverage. Reads were mapped to the hg19 reference genome sequence (GRCh37.p13; RefSeq assembly accession GCF_000001405.25) using BWA version 0.7.12 and biobambam2 version 2.0.27 was used to sort and mark duplicate

reads.^2,3^

Academic Forums

Two established academic fora were routinely consulted in cases where pathogenicity or actionability was challenging; 1) the monthly Copenhagen University Hospital Multidisciplinary Cardiogenetics Conference (CUHMCC) consisting of clinical geneticists, laboratory medicine specialists, cardiac pathologists, cardiologists, pediatric cardiologists, and 2) the National Network of Expertise for Cardiovascular Clinical Genetics (KardioGENet), consisting of clinical and laboratory geneticists from all Danish departments of Clinical Genetics and all Laboratory Medicine departments involved in cardiogenetic testing. For likely pathogenic variants in highly penetrant genes, CUHMCC was consulted and a strategy for clinical action was decided. For variants conferring a risk for a disorder with age-dependent penetrance (cardiomyopathies, aortopathies), the parents were offered testing for the variant and clinical exam for relevant phenotypical traits if carriers. For variants in genes that may present with symptoms or clinical findings in childhood (lipid disorders, long QT syndrome) the child was offered further clinical tests. For pathogenic variants in genes with incomplete and age-dependent penetrance (e.g. *SCN5A and PKP2*) CUHMCC and KardioGENet were consulted to find the balance between over- and undertreatment. It was then determined what the range of clinical workup (minimum, maximum) was deemed medically indicated, to inform the treating clinician in deciding future strategy for that family. This made it possible for the clinician to adjust the plan for a family according to specific wishes, while managing the family within national and multidisciplinary consensus.

The Danish health care setting

Denmark has a free universal health care system, where genetic counseling is offered through clinical genetics departments and in specialized clinics for inherited cardiac disorders or pediatric departments by specialized medical staff. Clinical diagnosis and management of hereditary cardiovascular and connective tissue disorders is based on national and European guidelines.^4–8^ Health information, such as diagnoses, bio-banked DNA, blood or tissue samples from deceased relatives can often be located through the extensive national registration systems and electronic medical records, if the patient has given consent. When cascade testing is encouraged in families, relatives are aided by medical professionals by providing information letters or referrals for relevant family members.

References

1 Byrjalsen A, Hansen TVO, Stoltze UK, Mehrjouy MM, Barnkob NM, Hjalgrim LL *et al.* Nationwide germline whole genome sequencing of 198 consecutive pediatric cancer patients reveals a high frequency of cancer prone syndromes. *PLoS Genet* 2020; **16**: 1–24.

2 Li H, Durbin R, Tischler G, Leonard S. Fast and accurate short read alignment with Burrows-Wheeler transform. *Source Code Biol Med* 2014; **9**: 1–18.

3 Tischler G, Leonard S. Biobambam: Tools for read pair collation based algorithms on BAM files. *Source Code Biol Med* 2014; **9**: 1–18.

4 Dansk Cardiologisk Selskab Holdningspapir: Familiær hyperkolesterolæmi. 2018 https://www.cardio.dk/familiaer-hyperkolesterolaemi-fh (accessed 23 Oct2023).

5 Dansk Cardiologisk Selskab: Arvelige hjertesygdomme hos børn. 2016 https://www.cardio.dk/arvelige-hjertesygdomme-hos-born (accessed 23 Oct2023).

6 Dansk Cardiologisk Selskab: Arvelige hjertesygdomme. 2013 https://www.cardio.dk/arvelige-hjertesygdomme2013 (accessed 23 Oct2023).

7 Dansk Selskab for Medicinsk Genetik guideline: Udredning og opfølgning af familiær aneurisme sygdom. 2020 https://dsmg.dk/kliniske-guidelines/dsmg-guidelines/ (accessed 23 Oct2023).

8 Aepc CC, Gregers B, Task W, Coordinator F, Behr ER, Kingdom U *et al.* 2022 ESC Guidelines for the management of V tachyarrhytmias and the prevention of sudden cardiac death death of the European Society of Cardiology ( ESC ). 2022; : 3997–4126.
