# Supplementary material for: Actionability and familial uptake following opportunistic genomic screening in a pediatric cancer cohort
Source: Eur J Hum Genet. 2024 May 13;32(7):846–57. doi: 10.1038/s41431-024-01618-7 (PMC11220050; doi:10.1038/s41431-024-01618-7)
Supplement: Supplementary file 2 — Table S1 [file 41431_2024_1618_MOESM2_ESM.docx]

Table S1: Variants in ACMG secondary findings v.2.0 gene list not disclosed to probands

| Gene | HGVS | HGVS (Protein) | Reason for not reporting* |
| --- | --- | --- | --- |
| *ATP7B* (NM_NM_000053.4) | c.2304dup | p.(Met769Hisfs*26) | only report biallelic |
| *ATP7B* (NM_NM_000053.4) | c.2804C>T | p.(Thr935Met) | only report biallelic |
| *ATP7B* (NM_NM_000053.4) | c.2972C>T | p.(Thr991Met) | only report biallelic |
| *ATP7B* (NM_NM_000053.4) | c.2998G>A | p.(Gly1000Arg) | only report biallelic |
| *ATP7B* (NM_NM_000053.4) | c.3207C>A | p.(His1069Gln) | only report biallelic |
| *CACNA1S* (NM_000069.2) | c.1234C>T | p.(Arg412Ter) | only report known pathogenic |
| *DSP* (NM_ NM_004415.3) | c.8188del | p.(Gln2730Serfs*16) | only report known pathogenic |

^*^  Kalia SS, Adelman K, Bale SJ *et al.* Recommendations for reporting of secondary findings in clinical exome and genome sequencing, 2016 update (ACMG SF v2.0): A policy statement of the American College of Medical Genetics and Genomics. *Genet Med* 2017; **19**: 249–255.
