# Supplementary material for: Actionability and familial uptake following opportunistic genomic screening in a pediatric cancer cohort
Source: Eur J Hum Genet. 2024 May 13;32(7):846–57. doi: 10.1038/s41431-024-01618-7 (PMC11220050; doi:10.1038/s41431-024-01618-7)
Supplement: Supplementary file 3 — Table S2 [file 41431_2024_1618_MOESM3_ESM.docx]

Table S2: Variant interpretation by ACMG classification criteria

| Family # | Gene | HGVS | HGVp | Classification criteria (ACMG guidelines)* | Variant classification (ACMG guidelines) * |
| --- | --- | --- | --- | --- | --- |
| Lipid Disorders | | | | | |
| 4 | *APOB* (NM_000384.2) | c.10580G>A | NP_000375.2: p.(Arg3527Gln) | PS3, PM2_sup, PP1, PP3 | PV |
| 10 | *APOB* (NM_000384.2) | c.10808A>G | NP_000375.2: p.(His3603Arg) | PM2_sup, BP4 | VUS |
| 13 | *APOB* (NM_000384.2) | c.11330C>A | NP_000375.2: p.(Ser3777Ter) | PVS1, PM2_sup | LPV |
| 15 | *APOB* (NM_000384.2) | c.10580G>A | NP_000375.2: p.(Arg3527Gln) | PS3, PM2_sup, PP1, PP3 | PV |
| 18 | *APOB* (NM_000384.2) | c.10134delG | NP_000375.2: p.(Gln3378Hisfs*4) | PVS1, PM2_sup | LPV |
| 12 | *LDLR* (NM_000527.4) | c.1359-1G>A | p.? | PVS1, PS3, PS4, PP1, PP3 | PV |
| 16 | *LDLR* (NM_000527.4) | c.1238C>T | NP_000518.1: p.(Thr413Met) | PS4, PM2_sup, PP1, PP3, PP4 | LPV |
| 19 | *LDLR* (NM_000527.4) | c.409G>A | NP_000518.1: p.(Gly137Ser) | PS4, PM2_sup, PP1, PP3 | LPV |
| Cardiomyopathy Disorders | | | | | |
| 1 | *DSG2* (NM_001943.4) | c.918G>A | NP_001934.2: p.(Trp306Ter) | PVS1, PS4, PM2_sup | PV |
| 3 | *MYBPC3* (NM_000256.3) | c.442G>A | NP_000247.2: p.(Gly148Arg) | PS3, PS4, PM2_sup, PP1 | LPV |
| 7 | *MYBPC3* (NM_000256.3) | c.3628-41_3628-17del | p.? | BS1 | BV |
| 14 | *MYBPC3* (NM_000256.3) | c.3628-41_3628-17del | p.? | BS1 | BV |
| 11 | *MYL2*(NM_000432.3) | c.403-1G>C | p.? | PM2_sup, PM3, PM4, PP1 | LPV |
| 17 | *PKP2* (NM_004572.4) | c.1643del | NP_004563.2: p.(Gly548Valfs*15) | PVS1, PS4, PM2_sup | PV |
| Arrythmia Disorders | | | | | |
| 2 | *KCNQ1* (NM_000218.2) | c.905C>T | NP_000209.2: p.(Ala302Val) | PS3, PS4, PM1, PM2_sup | PV |
| 8 | *KCNQ1* (NM_000218.2) | c.806G>T | NP_000209.2: p.(Gly269Val) | PM2_sup, PM5, PP3, PP4 | LPV |
| 5 | *SCN5A* (NM_198056.2) | c.611+1G>A | p.? | PVS1, PS4, PM2 | PV |
| Connective Tissue Disorders | | | | | |
| 9 | *COL3A1* (NM_000090.3) | c.2283+1G>A | p.? | PVS1, PM2_sup | LPV |
| 6 | *FBN1* (NM_000138.4) | c.6724C>T | NP_000129.3: p.(Arg2242Cys) | PS4_sup, PM1, PM2­_sup | VUS |

* Richards S, Aziz N, Bale S, Bick D, Das S, Gastier-Foster J *et al.* Standards and guidelines for the interpretation of sequence variants: A joint consensus recommendation of the American College of Medical Genetics and Genomics and the Association for Molecular Pathology. *Genet Med* 2015; **17**: 405–424.
