# Supplementary material for: Actionability and familial uptake following opportunistic genomic screening in a pediatric cancer cohort
Source: Eur J Hum Genet. 2024 May 13;32(7):846–57. doi: 10.1038/s41431-024-01618-7 (PMC11220050; doi:10.1038/s41431-024-01618-7)
Supplement: Supplementary file 4 — Figure S1 [file 41431_2024_1618_MOESM4_ESM.pptx]

## Slide 1
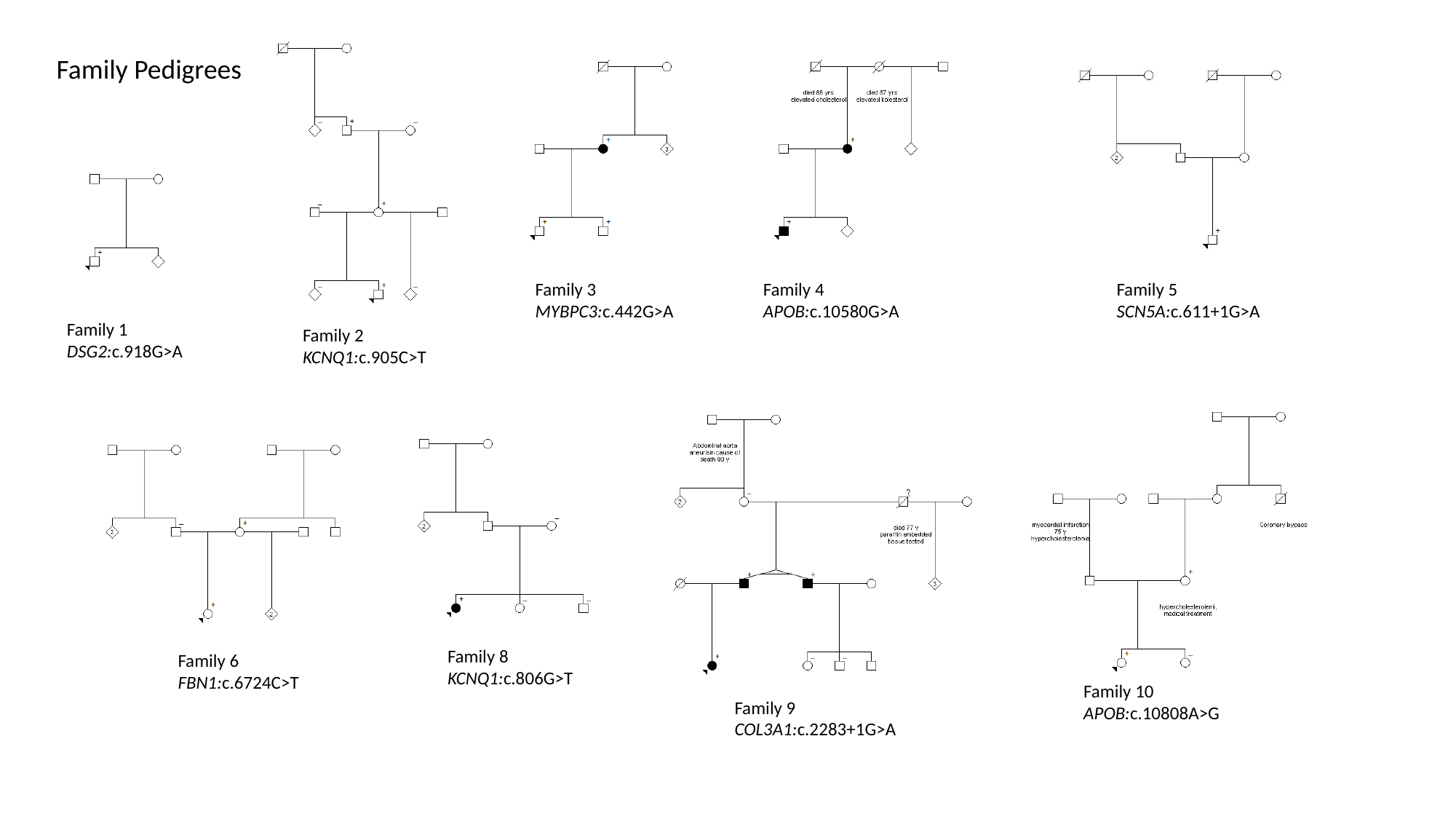

Family Pedigrees
Family 3 MYBPC3:c.442G>A
Family 4 APOB:c.10580G>A
Family 5 SCN5A:c.611+1G>A
Family 1
DSG2:c.918G>A
Family 2
KCNQ1:c.905C>T
Family 8 KCNQ1:c.806G>T
Family 6 FBN1:c.6724C>T
Family 10 APOB:c.10808A>G
Family 9 COL3A1:c.2283+1G>A

## Slide 2
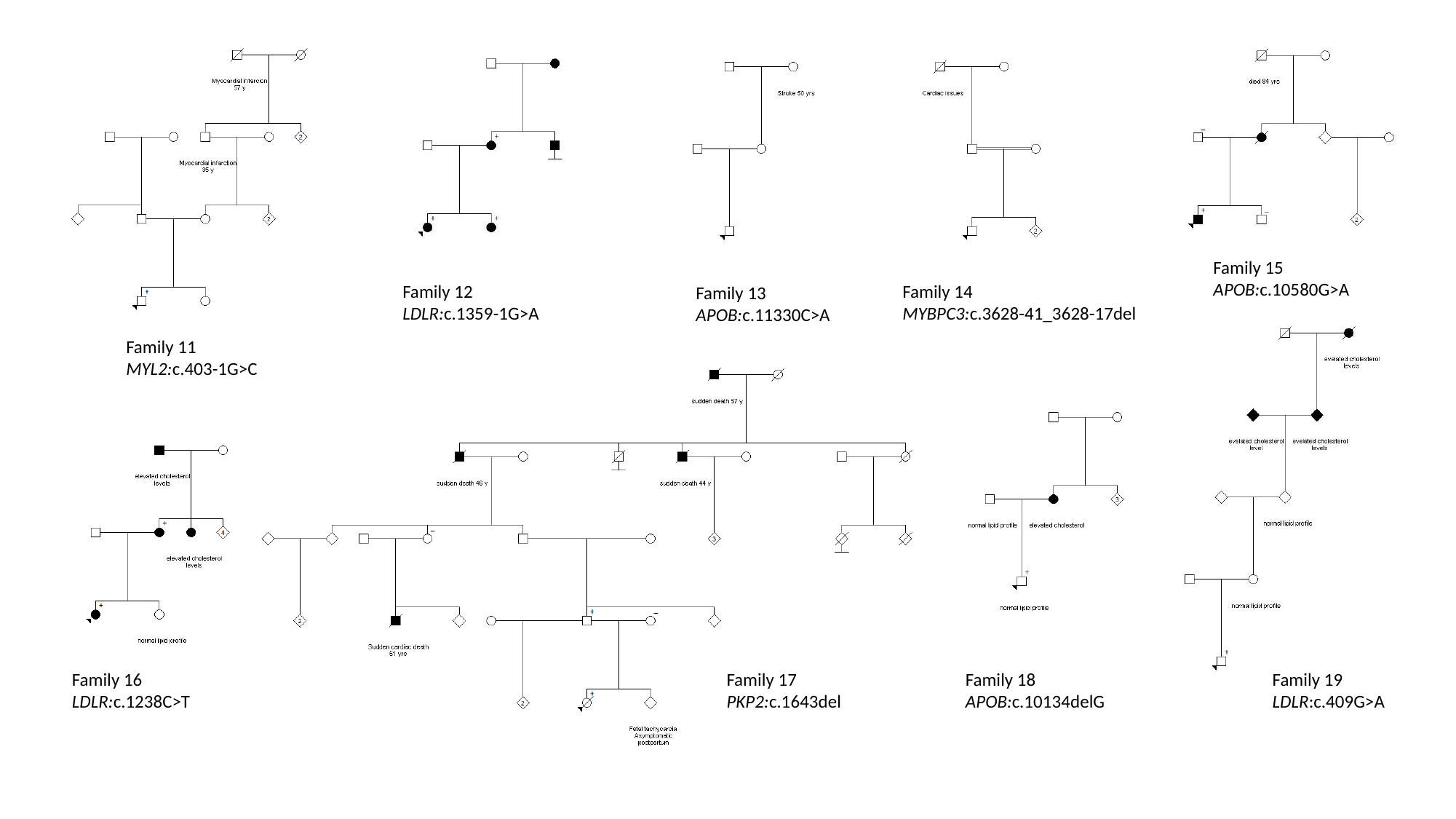

Family 15 APOB:c.10580G>A
Family 14
MYBPC3:c.3628-41_3628-17del
Family 12
LDLR:c.1359-1G>A
Family 13 APOB:c.11330C>A
Family 11
MYL2:c.403-1G>C
Family 19 LDLR:c.409G>A
Family 17 PKP2:c.1643del
Family 18 APOB:c.10134delG
Family 16 LDLR:c.1238C>T
