# Supplementary material for: Actionability and familial uptake following opportunistic genomic screening in a pediatric cancer cohort
Source: Eur J Hum Genet. 2024 May 13;32(7):846–57. doi: 10.1038/s41431-024-01618-7 (PMC11220050; doi:10.1038/s41431-024-01618-7)
Supplement: Supplementary file 5 — Supplementary Figure S1 Legend [file 41431_2024_1618_MOESM5_ESM.docx]

Supplementary Information:

Figure S1: Family pedigrees. In nine families, there was a relevant family history of cardiovascular disease (families 4, 9, 10, 12-16 and 19), but no diagnoses of a hereditary cardiovascular condition prior to the return of the secondary finding.
